# A Flexible Reduced Logarithmic-*X* Family of Distributions with Biomedical Analysis

**DOI:** 10.1155/2020/4373595

**Published:** 2020-02-20

**Authors:** Yinglin Liu, Muhammad Ilyas, Saima K. Khosa, Eisa Muhmoudi, Zubair Ahmad, Dost Muhammad Khan, G. G Hamedani

**Affiliations:** ^1^College of Pharmacy and Chemistry, Dali University, Dali City, Yunnan Province, China; ^2^Department of Statistics, University of Malakand, Dir (L), Chakdara, Khyber Pakhtunkhwa, Pakistan; ^3^Department of Statistics, Bahauddin Zakariya University, Multan, Pakistan; ^4^Department of Statistics, Yazd University, P.O. Box 89175-741, Yazd, Iran; ^5^Department of Statistics, Abdul Wali University Mardan, Mardan, Khyber Pakhtunkhwa, Pakistan; ^6^Department of Mathematical and Statistical Sciences, Marquette University, Milwaukee, WI 53201-1881, USA

## Abstract

Statistical distributions play a prominent role in applied sciences, particularly in biomedical sciences. The medical data sets are generally skewed to the right, and skewed distributions can be used quite effectively to model such data sets. In the present study, therefore, we propose a new family of distributions to model right skewed medical data sets. The proposed family may be named as a flexible reduced logarithmic-*X* family. The proposed family can be obtained via reparameterizing the exponentiated Kumaraswamy *G*-logarithmic family and the alpha logarithmic family of distributions. A special submodel of the proposed family called, a flexible reduced logarithmic-Weibull distribution, is discussed in detail. Some mathematical properties of the proposed family and certain related characterization results are presented. The maximum likelihood estimators of the model parameters are obtained. A brief Monte Carlo simulation study is done to evaluate the performance of these estimators. Finally, for the illustrative purposes, three applications from biomedical sciences are analyzed and the goodness of fit of the proposed distribution is compared to some well-known competitors.

## 1. Introduction

The statistical analysis and modeling of lifetime phenomena are essential in almost all areas of applied sciences, particularly, in biomedical sciences. A number of parametric continuous distributions for modeling lifetime data sets have been proposed in literature including exponential, Rayleigh, gamma, lognormal, and Weibull, among others. The exponential, Rayleigh, and Weibull distributions are more popular than the gamma and lognormal distributions since the survival functions of the gamma and the lognormal distributions cannot be expressed in closed forms and hence both require numerical integration to arrive at the mathematical properties. The exponential and Rayleigh distributions are commonly used in lifetime analysis. These distributions, however, are not flexible enough to counter complex forms of the data. For example, the exponential distribution is capable of modeling data with constant failure rate function, whereas the Rayleigh distribution offers data modeling with only increasing failure rate function. The Weibull distribution, also known as the super exponential distribution, is more flexible than the aforementioned distributions. The Weibull distribution offers the characteristics of both the exponential and Rayleigh distributions and is capable of modeling data with monotonic (increasing, decreasing, and constant) hazard rate function. Unfortunately, the Weibull distribution is not capable of modeling data with nonmonotonic (unimodal, modified unimodal, and bathtub-shaped) failure rate function. In some medical situations, for example, neck cancer, bladder cancer, and breast cancer, the hazard rate is shown to have unimodal or modified unimodal shape. The hazard rates for neck, bladder, and breast cancer recurrence after surgical removal have been observed to have unimodal shape. In the very initial phase, the hazard rate for cancer recurrence begins with a low level and then increases gradually after a finite period of time after the surgical removal until reaching a peak before decreasing. Another example of the unimodal shape is the hazard of infection with some new viruses, where it increases in the early stages from low level till it reaches a peak and then decreases; for detail, see [1]. In view of the importance of unimodal failure rate function in biomedical sciences, a series of papers have been appeared to propose new distributions capable of modeling medical data with unimodal failure rate function [2–8]. In the recent years, the researchers have shown a trend in proposing new families of distributions to obtain more flexible models. In this regard, [9] introduced the Marshall-Olkin generated (MOG) family by introducing an extra parameter to the Weibull distribution. The cumulative distribution function (cdf) of the MOG family is given by

(1) $$\begin{array}{c} G\left(x;\sigma ,\xi \right)=\frac{F\left(x;\xi \right)}{1-\left(1-\sigma \right)\left(1-F\left(x;\xi \right)\right)},\;\sigma >0,\;x\in \mathbb{R}, \end{array}$$

where *σ* is an additional parameter and *F*(*x*; *ξ*) is the cdf of the baseline model which may depend on the parameter vector *ξ*. [10] proposed another method of constructing new lifetime distributions known as alpha power transformation approach via cdf

(2) $$\begin{array}{c} G\left(x;\alpha_{1},\xi \right)=\frac{\alpha_{1}^{F\left(x;\xi \right)}-1}{\alpha_{1}-1},\;\alpha_{1}>0,\;\alpha_{1}\neq 1,\;x\in \mathbb{R}. \end{array}$$

Using (2), [10] and [11] introduced the alpha power exponential (APE) and alpha power transformed Weibull (APTW) distributions, respectively. We further carry this branch of distribution theory and introduce a new flexible class of distributions which can be used in modeling unimodal medical care data sets. Tahir and Corderio [12] proposed the exponentiated Kumaraswamy *G*-logarithmic (EKuGL) class of distributions given by the cdf:

(3) $$\begin{array}{c} G\left(x\right)=1-\;\frac{\log\left(1-\left(1-p\right)\left[1-{\left\{1-{\left(1-F{\left(x;\xi \right)}^{a}\right)}^{b}\right\}}^{\theta }\right]\right)}{\log\left(p\right)},\;x\in \mathbb{R}, \end{array}$$

where *a*, *b*, *θ* > 0 and *p* ∈ (0,1). For the EKuG-*L* family of distributions, the parametric space of *p* is restricted to (0, 1). Due to this relation, the EKuG-*L* family may not be flexible enough to counter complex forms of data. Furthermore, the EKuG-*L* family has four additional parameters. Note that the expression (3) is not true for *p*=1. Furthermore, due to the higher number of parameters, the estimation of the parameters as well as the computation of many distributional characteristics becomes very difficult. Therefore, in this paper, an attempt has been made to propose a more flexible class of distributions, called flexible reduced logarithmic-*X* (FRL-*X*) family via reparameterizing (3). The new family is introduced for *a*=*b*=*θ*=1 (to reduce the number of parameters to avoid the difficulties in computation of mathematical properties) and reparameterizing *p*=1+*σ* (to relax the upper limit of the parametric space of *p*), where *σ* > 0. In view of unrestricted upper bound, the proposed distribution would be quite flexible in modeling complex forms of data. Thus, the motivation for proposing the FRL-*X* family is to reduce the number of parameters as well as to relax the boundary conditions of the parametric values to bring more flexibility in the shape of the hazard rate function than the classical monotone behavior. Also, to improve the description which calls for complexity by adding the parameters in the class of distributions, this gives us more information about the behavior of the hazard rate function in the tail end. A random variable *X* is said to have the FRL-*X* distribution, if its cdf is given by

(4) $$\begin{array}{c} G\left(x;\sigma ,\xi \right)=1-\frac{\log\left\{1+\sigma -\sigma F\left(x;\xi \right)\right\}}{\log\left(1+\sigma \right)},\;\sigma >0,\;x\in \mathbb{R}, \end{array}$$

where *F*(*x*; *ξ*) is cdf of the baseline random variable depending on the parameter *ξ*, and *σ* is an additional parameter. The expression (4) is also true for *σ*=1. The probability density function (pdf) corresponding to (4) is given by

(5) $$\begin{array}{c} g\left(x;\sigma ,\xi \right)=\frac{\sigma f\left(x;\xi \right)}{\log\left(1+\sigma \right)\left\{1+\sigma -\sigma F\left(x;\xi \right)\right\}},\;x\in \mathbb{R}. \end{array}$$

The new pdf is most tractable when *F*(*x*; *ξ*) and *f*(*x*; *ξ*) have simple analytical expressions. Henceforth, a random variable *X* with pdf (5) is denoted by *X* ~ FRL − *X*(*x*; *σ*, *ξ*). Furthermore, for the sake of simplicity, the dependence on the vector of the parameters is omitted and *G*(*x*)=*G*(*x*; *σ*, *ξ*) will be used. Moreover, the key motivations for using the FRL-*X* family in practice are

1. A very simple and convenient method of adding an additional parameter to modify the existing distributions
2. To improve the characteristics and flexibility of the existing distributions
3. To introduce the extended version of the baseline distribution having closed forms for cdf, sf, and hrf
4. To provide better fits than the competing modified models
5. To introduce new distributions having nonmonotonic shaped hazard rate functions
6. To provide best fit to unimodal medical care data sets

The FRL-*X* family can also be obtained via reparameterizing the alpha logarithmic family (ALF) proposed by [13]. The cdf of the ALF family is given by

(6) $$\begin{array}{c} G\left(x;\sigma ,\xi \right)=1-\left(\frac{\log\left\{\alpha -\left(\alpha -1\right)F\left(x;\xi \right)\right\}}{\log\left(\alpha \right)}\right),\;\alpha >0,\;\alpha \neq 1,\;x\in \mathbb{R}. \end{array}$$

The problem with ALF family is that *α*=1, and consequently, the parametric space of *α* is restricted. The RFL-*X* addressed this problem via reparameterizing *α* as *α*=*σ*+1. The advantage of the FRL-*X* family over the ALF is that *σ*=1 acceptable, and its parametric space is not restricted. Furthermore, for *σ*=1, the FRL-*X* reduces to the logarithmic transformed family of [14] given by

(7) $$\begin{array}{c} G\left(x;\xi \right)=1-\frac{\log\left\{2-F\left(x;\xi \right)\right\}}{\log\left(2\right)},x,\xi \in \mathbb{R}. \end{array}$$

The survival function (sf) and hazard function of the FRL-*X* family are given, respectively, by

(8) $$\begin{array}{c} S\left(x;\sigma ,\xi \right)=\frac{\log\left\{1+\sigma -\sigma F\left(x;\xi \right)\right\}}{\log\left(1+\sigma \right)},\;x\in \mathbb{R},\\ h\left(x;\sigma ,\xi \right)=\frac{\sigma f\left(x;\xi \right)}{\left\{1+\sigma -\sigma F\left(x;\xi \right)\right\}\log\left\{1+\sigma -\sigma F\left(x;\xi \right)\right\}},\;x\in \mathbb{R}. \end{array}$$

The rest of this article is organized as follows. In Section 2, a special submodel of the proposed family is discussed. Some mathematical properties are obtained in Section 3. The characterizations results are presented in Section 4. Maximum likelihood estimates of the model parameters are obtained in Section 5. A comprehensive Monte Carlo simulation study is conducted in Section 6.  Section 7 is devoted to analyzing three real-life applications. Further framework is discussed in Section 8. Finally, concluding remarks are provided in the last section.

## 2. SubModel Description

This section offers a special submodel of the FRL-*X* family, called the flexible reduced logarithmic-Weibull (FRL-W) distribution. Let *F*(*x*; *ξ*) and *f*(*x*; *ξ*) be cdf and pdf of the two-parameter Weibull distribution given by *F*(*x*; *ξ*)=1 − *e*^−*γx*^*α*^^,  *x* ≥ 0,  *α*, *γ* > 0, and *f*(*x*; *ξ*)=*αγx*^*α*−1^*e*^−*γx*^*α*^^, respectively, where *ξ*=(*α*, *γ*). Then, the cdf of the FRL-W distribution has the following expression:

(9) $$\begin{array}{c} G\left(x\right)=1-\frac{\log\left\{1+\sigma -\sigma \left(1-e^{-\gamma x^{\alpha }}\right)\right\}}{\log\left(1+\sigma \right)},\;x\geq 0,\;\sigma ,\alpha ,\gamma >0. \end{array}$$

The density function corresponding (9) is given by

(10) $$\begin{array}{c} g\left(x\right)=\frac{\alpha \sigma \gamma x^{\alpha -1}e^{-\gamma x^{\alpha }}}{\log\left(1+\sigma \right)\left\{1+\sigma -\sigma \left(1-e^{-\gamma x^{\alpha }}\right)\right\}},\;x>0. \end{array}$$

**Figure 1:**
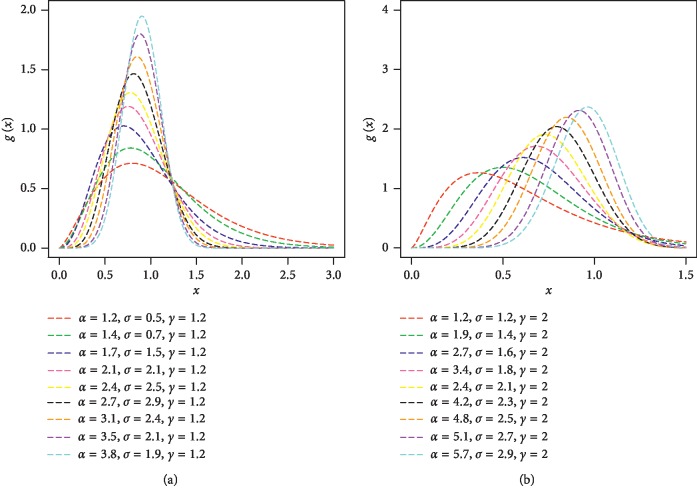
Different plots for the pdf of the FRL-W distribution.

Plots of the pdf of the FRL-W distribution are sketched in Figure 1 for selected values of the model parameters.

## 3. Basic Mathematical Properties

In this section, some statistical properties of the FRL-*X* family are derived.

### 3.1. Quantile Function

Let *X* be the FRL-*X* random variable with cdf (4), the quantile function of *X*, say *Q*(*u*), is given by

(11) $$\begin{array}{c} x=Q\left(u\right)=G^{-1}\left(u\right)=F^{-1}\left(\frac{1+\sigma -e^{\log\left(1+\sigma \right)\left(1-u\right)}}{\sigma }\right), \end{array}$$

where *u* ∈ (0,1). From the expression (11), it is clear that the FRL-*X* family has a closed form solution of its quantile function which makes it easier to generate random numbers.

### 3.2. Moments

Moments are very important and play an essential role in statistical analysis, especially in the applications. It helps to capture the important features and characteristics of the distribution (e.g., central tendency, dispersion, skewness, and kurtosis). The *r*^th^ moment of the FRL-*X* family is derived as

(12) $$\begin{array}{c} \mu_{r}^{/}=\int_{-\infty }^{\infty }x^{r}g\left(x;\sigma ,\xi \right)\text{d}x. \end{array}$$

Using (5) in (12), we have

(13) $$\begin{array}{c} \mu_{r}^{/}=\int_{-\infty }^{\infty }x^{r}\frac{\sigma f\left(x;\xi \right)}{\log\left(1+\sigma \right)\left\{1+\sigma -\sigma F\left(x;\xi \right)\right\}}\text{d}x,\\ \mu_{r}^{/}=\frac{1}{\log\left(1+\sigma \right)}\left(\frac{\sigma }{1+\sigma }\right)\int_{-\infty }^{\infty }x^{r}\frac{f\left(x;\xi \right)}{\left\{1-\left(\sigma F\left(x;\xi \right)/1+\sigma \right)\right\}}\text{d}x. \end{array}$$

Using the series representation

(14) $$\begin{array}{c} \frac{1}{1-x}=1+x+x^{2}+\cdots =\sum_{i=0}^{\infty }x^{i},\;\text{for }0<x<1. \end{array}$$

For *x*=(*σ*/1+*σ*)*F*(*x*; *ξ*) in (14), we arrive at

(15) $$\begin{array}{c} \frac{1}{1-\left(\sigma /1+\sigma \right)F\left(x;\xi \right)}=1+x+x^{2}+\cdots =\sum_{i=0}^{\infty }{\left(\frac{\sigma }{1+\sigma }\right)}^{i}F{\left(x;\xi \right)}^{i}. \end{array}$$

Using (15) in (13), we obtain

(16) $$\begin{array}{c} \mu_{r}^{/}=\frac{1}{\log\left(1+\sigma \right)}\sum_{i=0}^{\infty }{\left(\frac{\sigma }{1+\sigma }\right)}^{i+1}\eta_{r,i}, \end{array}$$

where *η*_*r*,*i*_=∫_−*∞*_^*∞*^*x*^*r*^*f*(*x*; *ξ*)*F*(*x*; *ξ*)^*i*^d*x*.

Furthermore, a general expression for the moment generating function (mgf) of the RFL-*X* family is given by

(17) $$\begin{array}{c} M_{x}\left(t\right)=\frac{1}{\log\left(1+\sigma \right)}\sum_{r,i=0}^{\infty }{\left(\frac{\sigma }{1+\sigma }\right)}^{i+1}\frac{t^{r}\eta_{r,i}}{r!}. \end{array}$$

### 3.3. Residual and Reverse Residual Life

The residual life offers wider applications in reliability theory and risk management. The residual lifetime of FRL-*X*, denoted by *R*_(*t*)_, is

(18) $$\begin{array}{c} R_{\left(t\right)}\left(x\right)=\frac{S\left(x+t\right)}{S\left(t\right)},\\ R_{\left(t\right)}\left(x\right)=\frac{\log\left\{1+\sigma -\sigma F\left(x+t;\xi \right)\right\}}{og\left\{1+\sigma -\sigma F\left(x;\xi \right)\right\}},\;x\in \mathbb{R}. \end{array}$$

Additionally, the reverse residual life of the FRL-*X* random variable, denoted by ${\bar{R}}_{\left(t\right)}$, is

(19) $$\begin{array}{c} {\bar{R}}_{\left(t\right)}=\frac{S\left(x-t\right)}{S\left(t\right)},\\ {\bar{R}}_{\left(t\right)}=\frac{\log\left\{1+\sigma -\sigma F\left(x-t;\xi \right)\right\}}{\log\left\{1+\sigma -\sigma F\left(x;\xi \right)\right\}},\;x\in \mathbb{R}. \end{array}$$

## 4. Characterization Results

This section is devoted to the characterizations of the FRL-*X* distribution based on a simple relationship between two truncated moments. It should be mentioned that for this characterization the cdf is not required to have a closed form. The first characterization result employs a theorem due to [15]; see Theorem 1 below. Note that the result holds also when the interval *H* is not closed. Moreover, as shown in [23], this characterization is stable in the sense of weak convergence.

**Theorem 1 .**

Let (*Ω*, *Ƒ*, *P*) be a given probability space and let *H*=[*d*, *e*] be an interval for some *d* < *e* (*d*=−*∞e*=*∞* might as well be allowed). Let *X*: *Ω*⟶*H* be a continuous random variable with the distribution function *G* and let *q*_1_ and *q*_2_ be two real functions defined on *H* such that

(20) $$\begin{array}{c} E\left(\left.q_{2}\left(X\right)\right|X\geq x\right)=E\left(\left.q_{1}\left(X\right)\right|X\geq x\right)\eta \left(x\right),\;x\in H, \end{array}$$

Is defined with some real function *η*. Assume that *q*_1_, *q*_2_ ∈ *C*^1^,  *η* ∈ *C*^2^ and *G* is twice continuously differentiable and strictly monotone function on the set *H*. Finally, assume that the equation *ηq*_1_=*q*_2_ has no real solution in the interior of *H*. Then G is uniquely determined by the functions *q*_1_, *q*_2_, and *η*, particularly

(21) $$\begin{array}{c} G\left(x\right)=\int_{a}^{x}C\left|\frac{\eta^{/}\left(u\right)}{\eta \left(u\right)q_{1}\left(u\right)-q_{2}\left(u\right)}\right|\;\exp\left(-s\left(u\right)\right)\text{d}u, \end{array}$$

where the function *s* is a solution of the differential equation *s*^/^(*u*)=*η*^/^*q*_1_/*ηq*_1_ − *q*_2_ and *C* is the normalization constant, such that ∫_*H*_*dF*=1.

**Remark 1 .**

The goal in Theorem 1 is to have *η* as simple as possible.

**Proposition 1 .**

Let*X* : *Ω*⟶*ℝ* be a continuous random variable and let *q*_1_(*x*)=1+*σ* − *σF*(*x*; *ξ*) and *q*_2_(*x*)=*q*_1_(*x*)*F*(*x*; *ξ*) for *x* ∈ *ℝ*. The random variable *X* has pdf (5) if and only if the function *η* defined in Theorem 1 is of the form

(22) $$\begin{array}{c} \eta \left(x\right)=\frac{1}{2}\left\{1+F\left(x;\xi \right)\right\},\;x\in \mathbb{R}. \end{array}$$

**Proof**

Let *X* be a random variable with pdf (5), then

(23) $$\begin{array}{c} \left(1-G\left(x\right)\right)E\left(q_{1}\left(X\right)\left|X\right.\geq x\right)=\frac{\sigma }{\log\left(1+\sigma \right)}\left\{1-F\left(x;\xi \right)\right\},\;x\in \mathbb{R},\\ \left(1-G\left(x\right)\right)E\left(q_{2}\left(X\right)\left|X\right.\geq x\right)=\frac{\sigma }{2\;\log\left(1+\sigma \right)}\left\{1-F{\left(x;\xi \right)}^{2}\right\},\;x\in \mathbb{R}, \end{array}$$

and finally

(24) $$\begin{array}{c} \eta \left(x\right)q_{1}\left(x\right)-q_{2}\left(x\right)=-\frac{q_{1}\left(x\right)}{2}\left(1-F\left(x;\xi \right)\right)>0,\;\text{for }x\in \mathbb{R}. \end{array}$$

Conversely, if *η* is given as above, then

(25) $$\begin{array}{c} s^{/'}\left(x\right)=\frac{\eta^{/}\left(x\right)q_{1}\left(x\right)}{\eta \left(x\right)q_{1}\left(x\right)-q_{2}\left(x\right)}=\frac{f\left(x;\xi \right)}{1-F\left(x;\xi \right)},\;x\in \mathbb{R}, \end{array}$$

and hence

(26) $$\begin{array}{c} s\left(x\right)=-\log\left\{1-F\left(x;\xi \right)\right\},\;x\in \mathbb{R}. \end{array}$$

Now, in view of Theorem 1, *X* has density (5).

**Corollary 1 .**

Let *X* : *Ω*⟶*ℝ* be a continuous random variable and let *q*_1_(*x*) be as in Proposition 1. The random variable *X* has pdf (5) if and only if there exist functions *q*_2_(*x*) and *η*(*x*) defined in Theorem 1 satisfying the following differential equation:

(27) $$\begin{array}{c} s^{/}\left(x\right)\frac{\eta^{/}\left(x\right)q_{1}\left(x\right)}{\eta \left(x\right)q_{1}\left(x\right)-q_{2}\left(x\right)}=\frac{f\left(x;\xi \right)}{\left[1-F\left(x;\xi \right)\right]},\;x\in \mathbb{R}. \end{array}$$

**Corollary 2 .**

The general solution of the differential equation in Corollary 1 is

(28) $$\begin{array}{c} \eta \left(x\right)={\left\{1-F\left(x;\xi \right)\right\}}^{-1}\left[-\int f\left(x;\xi \right){\left(q_{1}\left(x\right)\right)}^{-1}q_{2}\left(x\right)dx+D\right], \end{array}$$

where *D* is a constant. We like to point out that one set of functions satisfying the above differential equation is given in Proposition 1 with *D*=1/2. Clearly, there are other triplets (*q*_1_, *q*_2_, *η*) which satisfy conditions of Theorem 1.

## 5. Estimation

In this section, the method of maximum likelihood estimation is used to estimate the model parameters. Furthermore, the robustness is also discussed.

### 5.1. Maximum Likelihood Estimation

In this subsection, the maximum likelihood estimators (MLEs) of the parameters *σ* and *ξ* of RFL-*X* family from complete samples are derived. Let *X*_1_, *X*_2_,…, *X*_*k*_ be a simple random sample from RFL-*X* family with observed values *X*_1_, *X*_2_,…, *X*_*k*_. The log-likelihood function for this sample is

(29) $$\begin{array}{c} \log\;L\left(x;\sigma ,\xi \right)=n\;\log\left(\sigma \right)+\sum_{i=1}^{n}\log\;f\left(x_{i};\xi \right)-\log\left(\log\left(1+\sigma \right)\right)-\log\left\{1+\sigma -\sigma F\left(x_{i};\xi \right)\right\}. \end{array}$$

Obtaining the partial derivatives of (29), we have

(30) $$\begin{array}{c} \frac{\partial }{\partial \sigma }\log\;L\left(x;\sigma ,\xi \right)=\frac{n}{\sigma }-\frac{1}{\log\left(1+\sigma \right)\left(1+\sigma \right)}-\frac{1-F\left(x_{i};\xi \right)}{\left\{1+\sigma -\sigma F\left(x_{i};\xi \right)\right\}},\\ \frac{\partial }{\partial \xi }\log\;L\left(x;\sigma ,\xi \right)=\sum_{i=1}^{n}\frac{\partial f\left(x_{i};\xi \right)/\partial \xi }{f\left(x_{i};\xi \right)}+\sigma \frac{\partial F\left(x_{i};\xi \right)/\partial \xi }{\left\{1+\sigma -\sigma F\left(x_{i};\xi \right)\right\}}. \end{array}$$

Setting (∂/∂*σ*)log *L*(*x*; *σ*, *ξ*) and (∂/∂*ξ*)log *L*(*x*; *σ*, *ξ*) equal to zero and solving numerically these expressions simultaneously yields the MLEs of (*σ*, *ξ*).

### 5.2. M-Estimator as a Robust Estimation

Robust statistics are statistics with good performance for the data drawn from a wide range of probability distributions, especially for nonnormal distributions. Robust statistical approach has been developed for many common problems, such as estimating location, scale, and regression. One motivation is to produce statistical methods that are not unduly affected by outliers. Another motivation is to provide methods with good performance when there are small departures from the parametric distribution. For example, robust methods work well for mixtures of two normal distributions with different standard-deviations; under this model, nonrobust methods like a *t*-test work poorly. Historically, several approaches to robust estimation were proposed, including *R*-estimators and *L*-estimators. However, *M*-estimators now appear to dominate the field as a result of their generality, high breakdown point, and their efficiency. *M*-estimators are generalization of the maximum likelihood estimators (MLEs). What we try to do with MLE's is to maximize ∏_*i*=1_^*n*^*f*(*x*_*i*_) or, equivalently, minimize ∑_*i*=1_^*n*^−log(*f*(*x*_*i*_)) [16] proposed to generalize this to the minimization of ∑_*i*=1_^*n*^*ρ*(*x*_*i*_), where *ρ* is some function. MLEs are therefore special case of M-estimators. Minimizing ∑_*i*=1_^*n*^*ρ*(*x*_*i*_) can often be done by differentiating *ρ* and solving ∑_*i*=1_^*n*^*φ*(*x*_*i*_) where *φ*(*x*)=∂*ρ*(*x*)/∂*x*; for further detail, we refer the interested readers to [17, 18].

## 6. Monte Carlo Simulation Study

This section offers a comprehensive simulation study to assess the behavior of the MLEs. The FRL-*X* family is easily simulated by inverting (4). The expression (4) can be used to simulate any special submodel of the FRL-*X* family. Here, we consider the FRL-W distribution to assess the behavior of the MLEs of the proposed method. We simulate the FRL-W distribution for two sets of parameters (Set 1: *α* = 0.7, *σ* = 1.3, *γ* = 0.4, and Set 2:*α* = 1.4, *σ* = 1.6, *γ* = 1.2). The simulation is performed via the statistical software *R* through the command mle. The number of Monte Carlo replications made was 1000. For maximizing the log-likelihood function, we use the *L*-BFGS-*B* algorithm with optimum function. The evaluations of the estimators were performed via the following quantities for each sample size. The empirical mean squared errors (MSEs) are calculated using the *R* package from the Monte Carlo replications. The MLEs are determined for each piece of simulated data, say, $\left({\hat{\alpha }}_{i},{\hat{\gamma }}_{i},{\hat{\sigma }}_{i}\right)$ for *i* = 1,2,…, 1000; and the biases and MSEs are computed by

(31) $$\begin{array}{c} \text{Bias}\left(w\right)=\frac{1}{1000}\sum_{i=1}^{1000}\left({\hat{w}}_{i}-w\right),\\ \text{MSE}\left(w\right)=\frac{1}{1000}\sum_{i=1}^{1000}{\left({\hat{w}}_{i}-w\right)}^{2}. \end{array}$$

**Figure 2:**
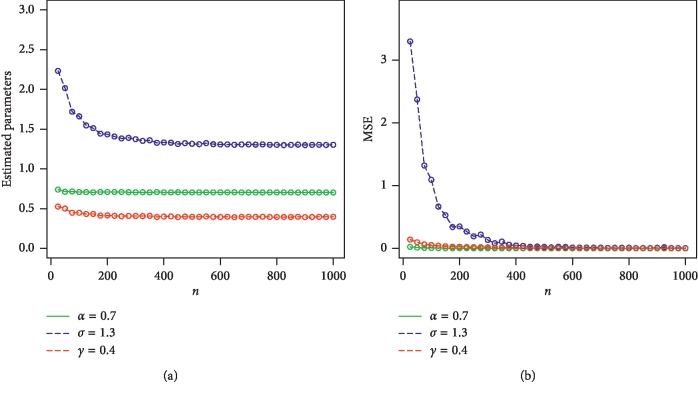
Plots of estimated parameters and MSEs for *α*=0.7, *σ*=1.3, and *γ*=0.4. (a) Plot of estimated parameters vs *n*. (b) Plot of MSE vs *n*.

**Figure 3:**
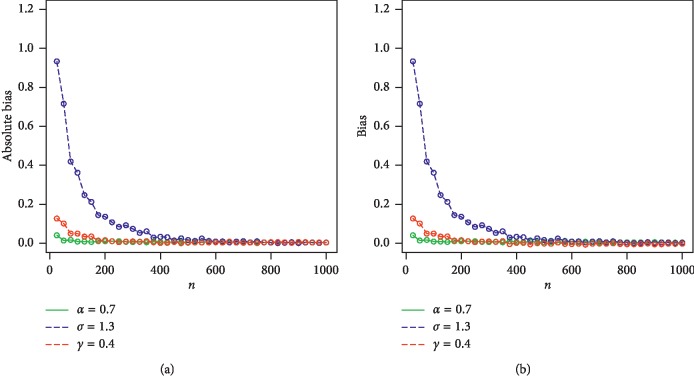
Plots of biases and absolute biases for *α*=0.7, *σ*=1.3, and *γ*=0.4. (a) Plot of absolute bias vs *n*. (b) Plot of bias vs *n*.

**Figure 4:**
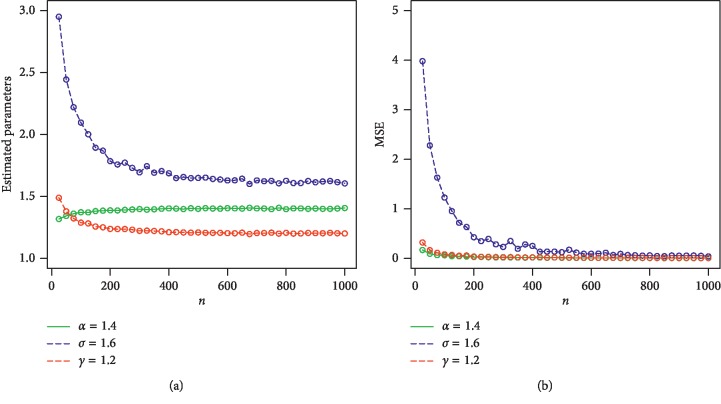
Plots of the parameters and MSEs for *α*=1.4, *σ*=1.6, and *γ*=1.2. (a) Plot of estimated parameters vs *n*. (b) Plot of MSEs vs *n*.

**Figure 5:**
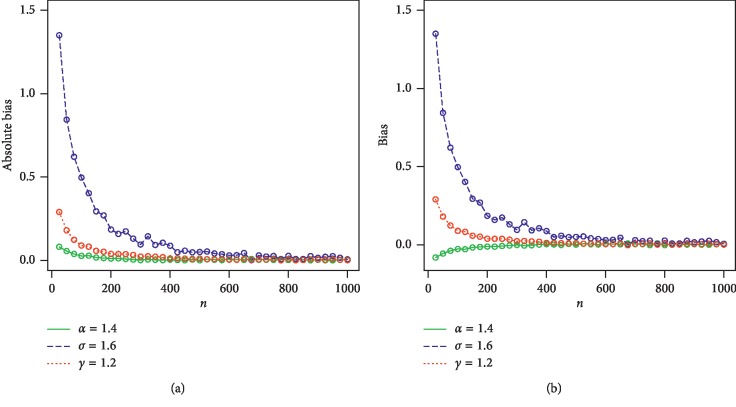
Plots of biases and absolute biases for *α*=1.4, *σ*=1.6, and *γ*=1.2. (a) Plot of absolute biases vs *n*. (b) Plot of biases vs *n*.

For *w*=*α*, *γ*, *β*, we consider the sample sizes at *n* = 25, 50, 100, 200, 400, 600, 800, 900, and 1000. The empirical results are given in Tables 1 and 2. Corresponding to Tables 1 and 2, the simulation results are graphically displayed in Figures 2–5. From the simulation results, we conclude that

- Biases for all parameters are positive
- The parameters tend to be stabilized
- Estimated biases decrease when the sample size *n* increases
- Estimated MSEs decay toward zero when the sample size *n* increases

## 7. Comparative Study

In this section, we illustrate the flexibility of the proposed model via three biomedical data sets. We also compare the proposed model with the other well-known models. The distribution functions of the competitive models are

- Weibull

(32) $$\begin{array}{c} G\left(x;\alpha ,\gamma \right)=1-e^{-\gamma x^{\alpha }},\;x\geq 0,\;\alpha ,\gamma >0. \end{array}$$

(ii) APTW distribution

(33) $$\begin{array}{c} G\left(x;\alpha_{1},\alpha ,\gamma \right)=\frac{\alpha_{1}^{\left(1-e^{-\gamma x^{\alpha }}\right)}-1}{\alpha_{1}-1},\;x\geq 0,\;\alpha_{1}\neq 1,\;\alpha ,\gamma >0. \end{array}$$

(iii) Marshall-Olkin Weibull (MOW) distribution

(34) $$\begin{array}{c} G\left(x;\alpha ,\gamma ,\sigma \right)=\frac{\left(1-e^{-\gamma x^{\alpha }}\right)}{\sigma +\left(1-\sigma \right)\left(1-e^{-\gamma x^{\alpha }}\right)},\;x\geq 0,\;\alpha ,\gamma ,\sigma >0. \end{array}$$

  To determine the optimum model, we compute Cramer–Von Messes (CM) test statistic, Anderson Darling (AD) test statistic, and Kolmogorov Simonrove (KS) test statistics with corresponding *p* values. These values are calculated as follows:
(iv) The AD test statistic

(35) $$\begin{array}{c} \text{AD}=-n-\frac{1}{n}\sum_{i=1}^{n}\left(2i-1\right)\left[\log\;G\left(x_{i}\right)+\log\left(1-\log\;G\left(x_{n-i+1}\right)\right)\right], \end{array}$$

  where *n* is the sample size and *x*_*i*_ is the *i*^th^ sample, calculated when the data is sorted in ascending order.
(v) The CM test statistic

(36) $$\begin{array}{c} \text{CM}=\frac{1}{12n}+{\sum_{i=1}^{n}\left[\frac{2i-1}{2n}+G\left(x_{i}\right)\right]}^{2}. \end{array}$$

(vi) The KS test statistic

(37) $$\begin{array}{c} \text{KS}=\sup\left[G_{n}\left(x\right)+G\left(x\right)\right], \end{array}$$

  where *G*_*n*_(*x*) is the empirical cdf, and sup_*x*_ is the supremum of the set of distances. A distribution with lower values of these measures is considered a good candidate model among the applied distributions for the underlying data sets. By considering these statistical tools, we observed that the FRL-W distribution provides the best fit compared to the other competitors since the values of all selected criteria of goodness of fit are significantly smaller for the proposed distribution.

### 7.1. Data 1: The Remission Times (in Months) of a Random Sample of 128 Bladder Cancer Patients

**Figure 6:**
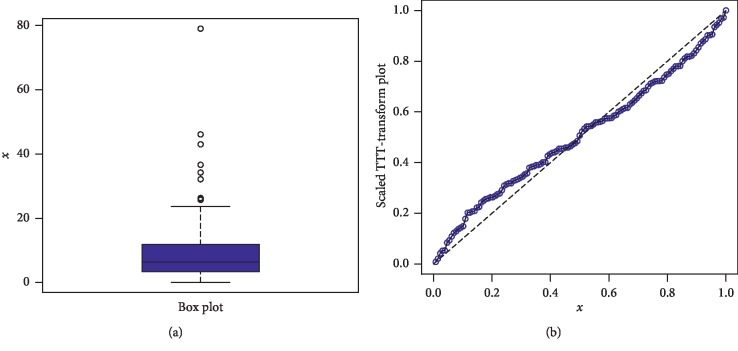
Box plot and Time Scale TTT plot of the first data set.

**Figure 7:**
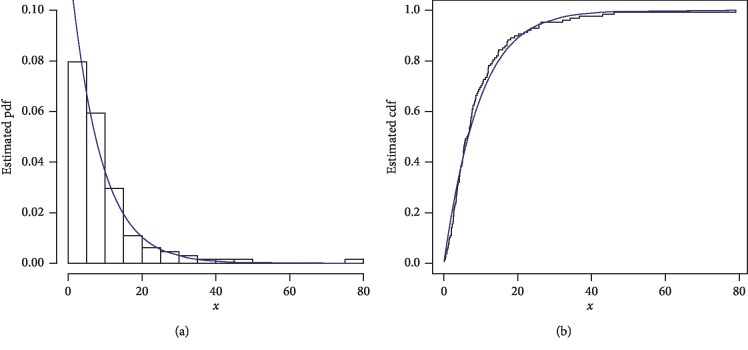
Estimated pdf and cdf of the FRL-W distribution for the first data set.

**Figure 8:**
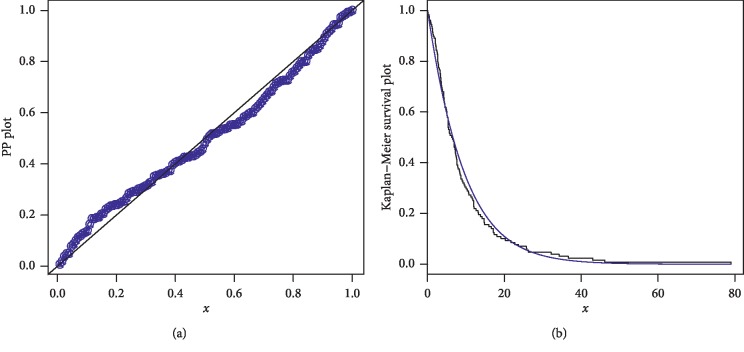
PP and Kaplan–Meier survival plots of the FRL-W distribution for the first data set.

The first data set represents the remission times (in months) of a random sample of 128 bladder cancer patients; see [19]. The FRL-W and the considered distributions are applied to this data set. The maximum likelihood estimates of the models for the analyzed data are presented in Table 3, whereas the goodness of fit measures of the proposed and other competitive models are provided in Table 4. Form Table 4, it is clear that the proposed distribution has lower values than the other models applied in comparison. The box plot and Time Scale TTT plot of the first data set are presented in Figure 6. The fitted pdf and cdf of the proposed model are plotted in Figure 7, whereas the PP and Kaplan–Meier survival plots of the proposed model for the first data set are sketched in Figure 8. From the Time Scale TTT plot (Figure 6), we can see that the first data set possess unimodal behavior. Also, from box plot in Figure 6, we can easily observe that the bladder cancer patient's data set is positively skewed. From Figure 7, it is clear that the proposed model fits the estimated pdf and cdf very closely. From Figure 8, we can easily detect that the proposed model is closely followed the PP-plot which is an empirical tool for finding a best candidate model.

### 7.2. Data 2: The Survival Times of Neck Cancer Patient Data

**Figure 9:**
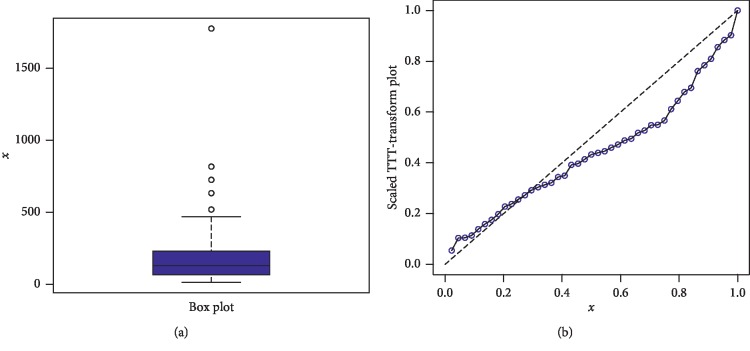
Box plot and Time Scale TTT plot of the second data set.

**Figure 10:**
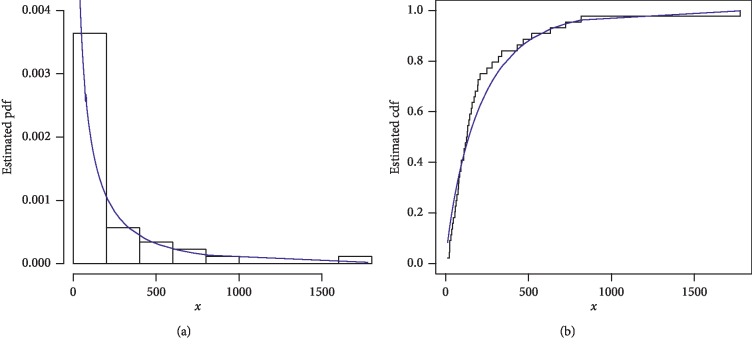
Estimated pdf and cdf of the FRL-W distribution for the second data set.

**Figure 11:**
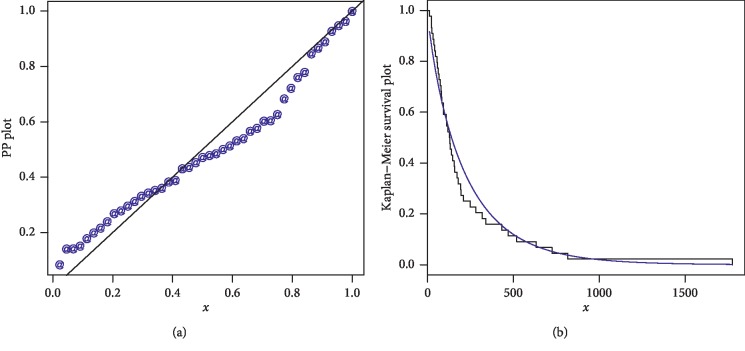
PP and Kaplan–Meier survival plots of the FRL-W distribution for the second data set.

The second data set consists of 44 observations taken from [20] represents the survival times of a group of patients suffering from head and neck cancer and treated using a combination of radiotherapy. This data set also used by [21]. We also applied the FRL-W and the other selected distributions to the second data set. Again, we observe that the proposed model outclasses the other competitors. Corresponding to data 2, the values of the model parameters are presented in Table 5. The analytical measures of the proposed and other competitive models are provided in Table 6. The box plot of the second data set and the corresponding Time Scale TTT plot of FRL-W are presented in Figure 9. The estimated pdf and cdf are sketched in Figure 10, which shows that proposed distribution fit the estimated pdf and cdf plots very closely, whereas the PP-Plot and Kaplan–Meier survival plots are presented in Figure 11. From the Time Scale TTT plot (Figure 9), we can see that the second data set possess the unimodal behavior. Also, from box plot in Figure 9, we can easily observe that the neck cancer data set is positively skewed. The proposed model also provides best fitting to the neck cancer data (see Table 6) and the proposed distribution fit the estimated pdf, cdf, and Kaplan–Meier survival plots very closely.

### 7.3. Data 3: The Guinea Pigs Infected Data

**Figure 12:**
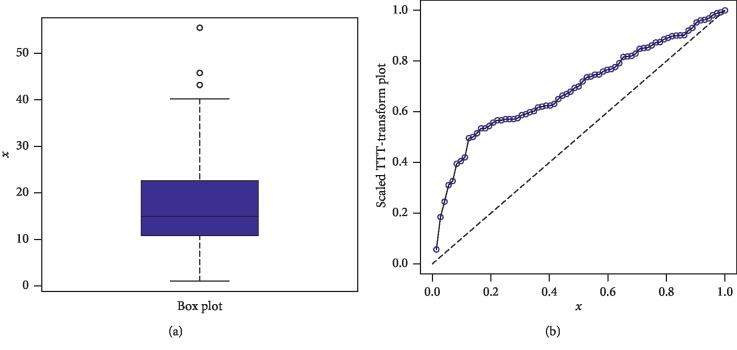
Box plot and Time Scale TTT plot of the third data set.

**Figure 13:**
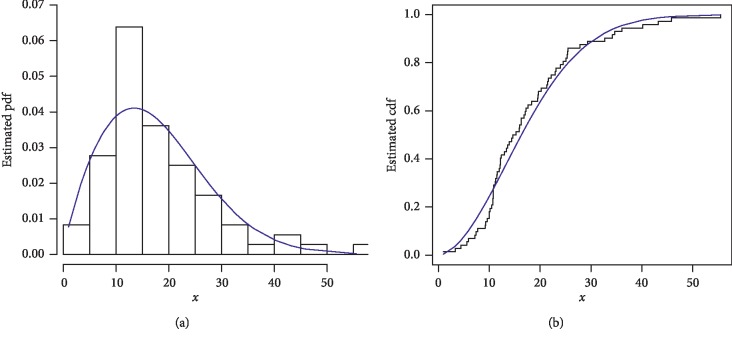
Estimated pdf and cdf of the FRL-W distribution for the third data set.

**Figure 14:**
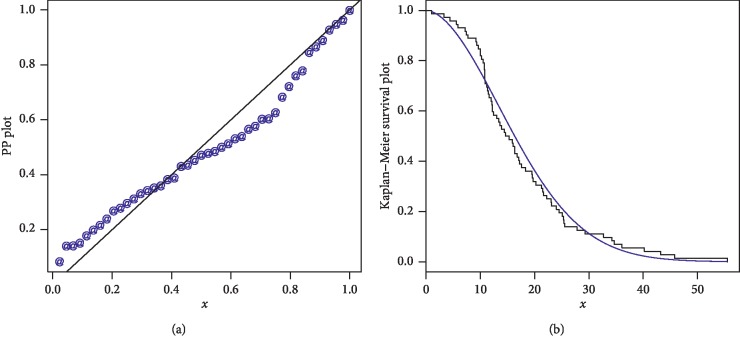
PP and Kaplan–Meier survival plots of the FRL-W distribution for the third data set.

The third data set consists of 72 observations taken from [22] representing the guinea pigs infected with virulent tubercle bacilli. Again, the FRL-W and other competitors are applied to this data set. Analyzing the third data set, we observe that the proposed model provides the better fit than the other competitors. Corresponding to data 3, the values of the model parameters are presented in Table 7. The analytical measures of the proposed and other competitive models are provided in Table 8. The box plot of the third data set and the corresponding Time Scale TTT plot of the FRL-W are presented in Figure 12. The estimated pdf and cdf are sketched in Figure 13, whereas the PP and Kaplan–Meier survival plots are provided in Figure 14. Figures 12–14 reveal that the FRL-W distribution provides the superior fits to the guinea pigs infected data.

## 8. Discussion and Future Frame Work

Statistical decision theory addresses the state of uncertainty and provides a rational framework for dealing with the problems of medical decision-making. The medical data sets are generally skewed to the right, and the positively skewed distributions are reasonably competitive when describing unimodal medical data. The traditional distributions are not flexible enough to counter complex forms of data such as medical sciences data having nonmonotonic failure rate function. In view of the importance of statistical distributions in applied sciences, a number of papers have been appeared in the literature aiming to improve the characteristics of the existing distributions. However, unfortunately the number of parameters has been increased and the estimation of the parameters and derivation of mathematical properties becomes complicated. Furthermore, due to the restricted parametric space, some distributions may not be flexible enough to provide adequate fit to many real data sets. To provide a better description of the medical sciences data, in this study, an attempt has been made to introduce a new family of statistical distributions by reducing the number of parameters and reparameterizing the existing distributions to relax the boundary conditions of the additional parameter. A special submodel of the proposed family offers the best fitting in data modeling with nonmonotonic hazard rate function. The maximum likelihood method is adopted to estimate the model parameters and a comprehensive Monte Carlo simulation study is done to evaluate the behavior of the estimators. To show the usefulness of the proposed method in medical sciences, three real-life examples are discussed. The very first example about bladder cancer patient data set is considered. The second data set represents the neck cancer data and third data set representing the guinea pigs infection. Analyzing these three real-life examples, it showed that the proposed model performs much better than the other competitive distributions. From the above discussion, it is obvious that the researchers are always in search of new flexible distributions. Therefore, to bring further flexibility in the proposed model, we suggest to introduce its extended versions. The proposed method can be extended by introducing a shape parameter to the model.

(i) A random variable *X* is said to follow the extended version of the FRL-*X* family, if its cdf is given by
  (38) $$\begin{array}{c} G\left(x;\theta ,\sigma ,\xi \right)=1-\frac{\log\left\{1+\sigma -\sigma F{\left(x;\xi \right)}^{\theta }\right\}}{\log\left(1+\sigma \right)},\;\theta ,\sigma ,\xi >0,\;x\in \mathbb{R}, \end{array}$$

where *θ* is the additional shape parameter. For *θ*=1, the expression (38) reduces to (4). The new proposal may be named as a flexible reduced logarithmic exponentiated-*X* (FRLE-*X*) family. For the illustrative purposes, one may consider its special case may be named as flexible reduced logarithmic exponentiated-Weibull (FRLE-W) distribution defined by the cdf:
  (39) $$\begin{array}{c} G\left(x;\theta ,\sigma ,\xi \right)=1-\frac{\log\left\{1+\sigma -\sigma {\left(1-e^{-\gamma x^{\alpha }}\right)}^{\theta }\right\}}{\log\left(1+\sigma \right)},\;x\geq 0,\;\theta ,\sigma ,\xi >0. \end{array}$$

Due to the introduction of the of additional shape parameter, the suggested extension may be much flexible in modeling data in medical sciences and other related fields.

(ii) Another extension of the FRL-*X* family is given by

(40) $$\begin{array}{c} G\left(x;\eta ,\sigma ,\xi \right)=1-{\left(\frac{\log\left\{1+\sigma -\sigma F\left(x;\xi \right)\right\}}{\log\left(1+\sigma \right)}\right)}^{\eta },\;\eta ,\sigma ,\xi >0,\;x\in \mathbb{R}, \end{array}$$

  where *η* is the additional shape parameter. For *η*=1 the expression (40) reduces to (4). The model defined in (40) may be named as the extended flexible reduced logarithmic-*X* (EFRL-*X*) family.

(iii) Another generalized version of the FRL-*X* can be introduced via

(41) $$\begin{array}{c} G\left(x;\eta ,\theta ,\sigma ,\xi \right)=1-{\left(\frac{\log\left\{1+\sigma -\sigma F{\left(x;\xi \right)}^{\theta }\right\}}{\log\left(1+\sigma \right)}\right)}^{\eta },\;\eta ,\theta ,\sigma ,\xi >0,\;x\in \mathbb{R}, \end{array}$$

where *θ* and *η* are the additional shape parameters. Clearly, for *η*=1, the expression (41) reduces to (38). For *θ*=1, the expression (41) reduces to (40), whereas for *θ*=*η*=1, the expression (41) reduces to (4). The model introduced in (41) may be named as the extended flexible reduced logarithmic exponentiated-*X* (EFRLE-*X*) family.

## 9. Concluding Remarks

In this study, we introduced a new family of continuous distributions called the flexible reduced logarithmic-*X* family. Some mathematical properties of the proposed family are obtained. The maximum likelihood method used to estimate the unknown model parameters. Three applications to the real-life medical data sets are given to illustrate empirically the flexibility of the proposed model. The comparison of the proposed method is made to some well-known lifetime distributions such as Weibull, Marshall-Olkin Weibull, and alpha power transformed Weibull distributions. The comparison is made on the basis of well-known goodness of fit measures including Cramer–Von Messes test statistic, Anderson Darling test statistic, and Kolmogorov–Simonrove test statistics with corresponding *p* values. Empirical findings indicate that the proposed model provide better fits than the other well-known competitive models.

## Figures and Tables

**Table 1:** Simulation results for the FRL-W distribution.

*n*	Set 1: *α*=0.7, *σ*=1.3, and *γ*=0.4.			
	Parameters	MLEs	MSEs	Biases
25	*α*	0.73230	0.02214	0.03230
	*σ*	2.27575	3.38670	0.97575
	*γ*	0.55680	0.18358	0.15680

50	*α*	0.72369	0.03247	0.02369
	*σ*	2.02097	2.45251	0.72097
	*γ*	0.50925	0.14881	0.10925

100	*α*	0.71358	0.00752	0.01358
	*σ*	1.65810	1.07306	0.35810
	*γ*	0.44796	0.05757	0.04796

200	*α*	0.70610	0.00352	0.00610
	*σ*	1.43857	0.32454	0.13857
	*γ*	0.41998	0.02678	0.01998

400	*α*	0.70723	0.00154	0.00723
	*σ*	1.33560	0.06391	0.03560
	*γ*	0.40108	0.01086	0.00108

600	*α*	0.70439	0.00079	0.00439
	*σ*	1.30650	0.01328	0.00650
	*γ*	0.39626	0.00504	-0.00373

800	*α*	0.70437	0.00054	0.00437
	*σ*	1.30093	0.00771	0.00093
	*γ*	0.39418	0.00343	-0.00581

900	*α*	0.70369	0.00046	0.00369
	*σ*	1.30177	0.00623	0.00177
	*γ*	0.39688	0.00316	-0.00311

1000	*α*	0.70168	0.00032	0.00168
	*σ*	1.30632	0.00533	0.00632
	*γ*	0.40068	0.00248	0.00068

**Table 2:** Simulation results for the FRL-W distribution.

*n*	Set 2: *α*=1.4, *σ*=1.6, and *γ*=1.2.			
	Parameters	MLEs	MSEs	Biases
25	*α*	1.31799	0.15082	−0.08200
	*σ*	2.88652	3.78920	1.28652
	*γ*	1.49688	0.30417	0.29688

50	*α*	1.32352	0.10232	−0.07647
	*σ*	2.53513	2.56035	0.93513
	*γ*	1.39342	0.18134	0.19342

100	*α*	1.36510	0.05251	−0.03489
	*σ*	2.03725	0.98667	0.43725
	*γ*	1.30355	0.08545	0.10355

200	*α*	1.38677	0.03109	−0.01322
	*σ*	1.84218	0.60026	0.24218
	*γ*	1.24274	0.03853	0.04274

400	*α*	1.40246	0.01610	0.00246
	*σ*	1.65938	0.18038	0.05938
	*γ*	1.21117	0.01882	0.01117

600	*α*	1.39853	0.01378	−0.00146
	*σ*	1.64008	0.09977	0.04008
	*γ*	1.20870	0.01429	0.00870

800	*α*	1.40399	0.00915	0.00399
	*σ*	1.61715	0.06442	0.01715
	*γ*	1.20592	0.01063	0.00592

900	*α*	1.40783	0.00784	0.00783
	*σ*	1.60754	0.05594	0.00753
	*γ*	1.20124	0.00877	0.00123

1000	*α*	1.40750	0.00765	0.00750
	*σ*	1.60425	0.04333	0.00425
	*γ*	1.19986	0.00839	0.00013

**Table 3:** Estimated values of the parameters with standard errors (in parentheses) of the competing models.

Dist.	$\hat{\alpha }$	$\hat{\gamma }$	$\hat{\sigma }$	$\hat{\lambda }$	${\hat{\alpha }}_{1}$
FRL-W	1.3269 (0.0815)	0.003 (0.0005)	12.978 (3.4361)		
Weibull	1.047 (0.0675)	0.093 (0.0190)			
APTW	0.014 (0.0865)	0.016 (0.0064)			0.014 (0.0216)
MOW	1.2684 (0.1308)	0.877 (0.5205)	11.829 (11.2869		

**Table 4:** Estimated values of the proposed and other competitive models.

Dist.	CM	AD	KS	*p* value
FRL-W	0.026	0.168	0.041	0.981
Weibull	0.131	0.786	0.069	0.558
APTW	0.042	0.255	0.045	0.949
MOW	0.150	0.884	0.075	0.451

**Table 5:** Estimated values of parameters of the proposed and other competitive models.

Dist.	$\hat{\alpha }$	$\hat{\gamma }$	$\hat{\sigma }$	${\hat{\alpha }}_{1}$
FRL-W	0.832 (0.1033)	0.013 (0.0112)	0.511 (0.0093)	
Weibull	0.940 (0.2755)	0.060 (0.0278)		
APTW	1.012 (0.1532)	0.003 (0.0044)		0.426 (0.5078)
MOW	0.908 (0.07328)	0.037 (0.0105)	1.029 (1.0359)	

**Table 6:** Estimated values of the parameters with standard errors (in parentheses) of the competing models.

Dist.	CM	AD	KS	*p* value
FRL-W	0.075	0.627	0.093	0.871
Weibull	0.141	0.823	0.130	0.406
APTW	0.121	0.713	0.107	0.653
MOW	0.108	0.704	0.905	0.721

**Table 7:** Estimated values of the parameters with standard errors (in parentheses) of the competing models.

Dist.	$\hat{\alpha }$	$\hat{\gamma }$	$\hat{\sigma }$	${\hat{\alpha }}_{1}$
FRL-W	1.799 (0.127)	0.423 (0.002)	0.005 (1.418)	
Weibull	1.814 (0.079)	0.004 (0.001)		
APTW	1.856 (0.077)	0.003 (0.001)		0.856 (1.048)
MOW	1.037 (0.279)	0.126 (0.152)	8.461 (9.153)	

**Table 8:** Goodness of fit measures of the proposed and other competitive models.

Dist.	CM	AD	KS	*p* value
FRL-W	0.130	0.705	0.081	0.438
Weibull	0.161	0.952	0.097	0.500
APTW	0.159	0.940	0.099	0.473
MOW	0.192	1.108	0.120	0.247

## Data Availability

This work is mainly a methodological development and has been applied on secondary data related cancer patients, but if required, data will be provided.
